# Recent Advances in Methamphetamine Neurotoxicity Mechanisms and Its Molecular Pathophysiology

**DOI:** 10.1155/2015/103969

**Published:** 2015-03-12

**Authors:** Shaobin Yu, Ling Zhu, Qiang Shen, Xue Bai, Xuhui Di

**Affiliations:** ^1^Department of Neurology, The Third Hospital of Hebei Medical University, Shijiazhuang 050051, China; ^2^Department of Neurology, Hospitals of The Armed Police Forces in Hebei, Shijiazhuang 050051, China

## Abstract

Methamphetamine (METH) is a sympathomimetic amine that belongs to phenethylamine and amphetamine class of psychoactive drugs, which are widely abused for their stimulant, euphoric, empathogenic, and hallucinogenic properties. Many of these effects result from acute increases in dopamine and serotonin neurotransmission. Subsequent to these acute effects, METH produces persistent damage to dopamine and serotonin release in nerve terminals, gliosis, and apoptosis. This review summarized the numerous interdependent mechanisms including excessive dopamine, ubiquitin-proteasome system dysfunction, protein nitration, endoplasmic reticulum stress, p53 expression, inflammatory molecular, D_3_ receptor, microtubule deacetylation, and HIV-1 Tat protein that have been demonstrated to contribute to this damage. In addition, the feasible therapeutic strategies according to recent studies were also summarized ranging from drug and protein to gene level.

## 1. Introduction

Methamphetamine (METH) is a kind of highly addictive psychostimulant drug that principally affects the monoamine neurotransmitter systems of the brain and results in feelings of alertness, increasing energy, and euphoria [1]. The compound was first synthesized from ephedrine in 1893 by the Japanese scientist Nagai Nagayoshi. In 1919, Akira Ogata synthesized crystallized METH by reducing ephedrine using red phosphorous and iodine, providing the basis for production of the drug on a larger scale [2]. In 1971, METH was restricted by US law, although oral METH (Ovation Pharmaceuticals) continues to be used today in the USA as a second-line treatment for a number of medical conditions, including attention deficit hyperactivity disorder (ADHD) and refractory obesity [3].

METH belongs to phenethylamine and amphetamine class of psychoactive drugs. It is an additive pharmacological psychostimulant of the central nervous system (CNS) which results in stimulating excessive dopaminergic transmission in the brain [4]. Ten percent of METH becomes biologically available within ten minutes of smoke inhalation, due to its high lipophilic nature [5]. METH generates an imbalance in the release and reuptake of dopamine, norepinephrine, and epinephrine producing intense euphoria followed by hours of stimulation, excitation, and alertness [6]. High doses of the METH can damage brain dopamine neurones in experimental animal studies [7–9]. However, it has been speculated that even low doses used clinically in psychiatry might cause brain damage [10]. Further, an epidemiological study revealed increased risk of development of Parkinson's disease in hospitalized patients with METH use disorders [11]. However, METH has been indiscriminately used given its high potential for abuse and addiction; this has negatively impacted the public health landscape at multiple levels [12].

The present research focuses on not only understanding the acute effects of euphoria feelings but also the long-term consequences of their abuse which are rapidly emerging and include evidence of brain injury and neurotoxicity [13]. Although numerous studies have illustrated the deleterious effects of METH on various components of the nervous system, precise cellular and biochemical mechanisms remain largely unknown. This review will highlight the underlying mechanisms associated with the neurotoxicity of METH and discuss the consequences associated with the neuronal damage produced by the METH. The understanding of the mechanisms involved in METH neurotoxicity could lead to the discovery of new strategies to prevent or counter neurotoxic and neurodegenerative processes.

## 2. Neurotoxicity of Methamphetamine

### 2.1. Acute Effects of High Dose

METH treatment causes acute increases in both dopamine (DA) and serotonin (5HT) release [13]. Secondary to increases in extracellular DA, METH also causes acute increases in striatal glutamate as a result of D_1_ DA receptor-mediated disinhibition of corticostriatal glutamate release [14]. Subsequent to the acute effects of exposure, METH produces long-term damage to dopaminergic and serotonergic axon terminals in the striatum, hippocampus, and prefrontal cortex [15]. Neurochemical markers of this toxicity include decreases in the expression of tyrosine and tryptophan hydroxylase, the rate limiting enzymes for DA and 5HT, respectively, as well as decreases in DA and 5HT tissue content and decreases in DAT and SERT expression [13].

### 2.2. Long-Term Damage of Low Dose

METH exposure results in long-term damage to the dopamine system in both human METH abusers and animal models. Chronic use of METH is often associated with cognitive deficits ranging from impaired impulse control, attentional problems, working memory, and decision making to motor coordination, including inhibitory control [52–55], which do not display classic Parkinsonian motor impairments. However, chronic users of METH are at higher risk for developing Parkinson's disease (PD) than nonusers [11, 56]. Other consequences of long-term METH abuse include a partial, but persistent, loss of DA and 5HT systems in multiple brain areas, such as striatum, cortex, and hippocampus [53, 57, 58]. Those could be the reasons of METH-induced partial monoamine toxicity [59].

### 2.3. Gliosis of METH Use

Gliosis is a natural reactive process of glial cells (astrocytes and microglia) to brain injury, damage, infection, or disturbed homeostasis. It is characterized in part by increased expression of glial-specific proteins and morphologically hypertrophied cell body and processes and, in some cases, also cell proliferation and migration [60]. The functions of gliosis following brain injury and whether the response is helpful or detrimental continue to be debated [61–63]. Some attention therefore has been focused on whether brain of METH users displays signs of either microgliosis or astrogliosis. Although these cellular changes were not equaling brain damage, which is typically observed following brain injury in general [64, 65], and primarily in the dopamine-rich striatum in brain of experimental animals exposed to high doses of METH [8, 66, 67], the latest research suggested that some astrocytic “disturbance” had occurred, which might in principle be related to METH neurotoxicity or to a neuroplastic remodeling process [68]. However, there is a debate about whether the brain gliosis is a characteristic of chronic METH use in the human or not.

### 2.4. Cell Death Caused by METH

Though not as extensively studied, there is also supporting evidence that METH may produce cell death, in addition to damaging DA and 5HT terminals. Increase in terminal deoxynucleotidyl transferase dUTP nick end labeling (TUNEL) has been reported after exposure to METH in the prefrontal cortex and striatum [69, 70]. This cell death has been identified in different subpopulations of GABA interneurons. Mitochondrial damage and endoplasmic reticulum stress have been associated with this METH-induced apoptosis [70]. Specifically, METH has been shown to produce apoptosis through increases in caspase-3 activity and the Fas/FasL cell death pathways [71]. METH also produces DNA damage and alterations in the expression of Bcl-2 related genes, which may contribute to GABA interneuron cell death [38, 72]. For years, several molecular mechanisms have been proposed to account for neurotoxic properties of METH, including oxidative stress and apoptosis in dopaminergic cell lines [73]. Recently, an increasing number of studies have reported that autophagy, a self-degradative process, also plays a role in the process of METH-induced cell injury [74, 75].

### 2.5. METH and HIV-1

There are approximately 10–15% of human immunodeficiency virus-1 (HIV-1) patients reporting METH use in the United States [4]. Risky behavior accompanies strong neurological impulses associated with METH abuse resulting in the high prevalence of METH users who acquire HIV-1 infection [76, 77]. On the other hand, clinical research describes that individuals infected with HIV-1 actively participate in METH abuse [78, 79]. Neurotoxic outcomes of METH abuse and HIV-1 CNS infection include, but are not limited to, brain hyperthermia, release of inflammatory mediators and reactive oxygen species (ROS), excitotoxicity, and astrogliosis [71, 80].

The combination of METH abuse and HIV infection may lead to substantial alterations in DA neuron functioning. Although the responsible mechanisms of HIV-1 CNS toxicity have not been well defined, neurotoxic viral proteins, such as Tat, released from infected cells, may be involved. Tat is a nonstructural viral protein that is necessary for viral replication. It is actively released* in vitro* by infected lymphoid and glial cells [16]. Intrastriatal injections of Tat have been shown to damage both efferent and afferent projections of the striatum [17], including nigrostriatal DA neurons [18].

Tat has been shown both* in vitro *and* in vivo* to lead to the production of ROS or oxidative damage [19]. The oxidative damage produced by each compound could be amplified when both toxins are present together. Another factor that could be adding to the oxidative stress is that the hyperthermia produced by the METH treatments could be adding to the metabolic stress induced by Tat and thus increase its toxicity. DNA-binding activities of NF-*κ*B, AP-1, and CREB in the frontal cortex and hippocampus were more pronounced in mice injected with Tat plus METH compared to the effects of Tat or METH alone. Intercellular adhesion molecule-1 gene expression was also upregulated in a synergistic manner in cortical, striatal, and hippocampal regions in mice which received injections of Tat combined with METH compared to the effects of these agents alone [81]. These indicated that Tat enhances METH-induced reductions in striatal DA release and content, possibly in a synergistic manner, and suggest that METH abusers infected with HIV may be at increased risk for basal ganglia dysfunction. Tat and METH can cross-amplify their cellular effects, leading to alterations of redox-regulated inflammatory pathways in the brain. Such synergistic proinflammatory stimulation may have significant implications in HIV-infected patients who abuse drugs [82].

## 3. Mechanisms of Neurotoxicity

Various hypotheses regarding the mechanism responsible for METH-induced neurotoxicity have been proposed, including hyperthermia, glutamate release, reactive oxygen species, reactive nitrogen species, apoptosis-related molecules, and dopamine quinone [7, 82]. It is likely that interactions between these factors initiate METH-induced neurotoxicity.

### 3.1. Excessive Dopamine

METH treatment produces sustained reduction in striatal dopamine levels, persistent loss of dopaminergic nerve terminals, reduced activity of tyrosine hydroxylase, and a pronounced loss of dopamine transporter. Additional evidence suggests that enhanced oxidative stress is a critical contributor to MATH-induced neurotoxicity [20–22]. The excessive release of DA induced by METH has been linked to increased production of reactive oxygen species. One factor that has been heavily implicated in this METH-induced damage to the dopaminergic system is the activation of D1 DA receptors. Even when METH-induced hyperthermia is maintained, the coadministration of a D1 DA receptor antagonist protects against METH-induced neurotoxicity, strongly suggesting that D1 DA receptors play an important role in METH-induced neurotoxicity apart from the mitigation of METH-induced hyperthermia [83]. Although it is well known that MATH causes DA terminal degeneration, accumulating evidence indicates that METH causes injury to cell bodies in diverse brain regions [84, 85].

### 3.2. Ubiquitin-Proteasome System (UPS) Dysfunction

The two major intracellular degradation systems in eukaryotic cells are the ubiquitin-proteasome system (UPS) and the autophagy lysosome (ALS) pathway. Impaired protein degradation has been implicated in the cellular toxicity and eventual degenerative processes in chronic neurodegenerative disorders including Parkinson's disease, Alzheimer's disease, Huntington's disease, and other related proteinopathies [25]. UPS primarily degrades short lived and cytosolic proteins. On the other hand, macroautophagy degrades soluble proteins, large aggregates, and organelles in the cytoplasm and is a highly conserved bulk degradation system in eukaryotes [26]. Recent studies indicate that METH-induced neurotoxicity is associated with the formation of ubiquitin-positive aggregates and multilamellar bodies, suggesting that induction of autophagy may constitute a cytoprotective response after METH treatment [27, 28]. Likewise, ubiquitin-positive proteinaceous inclusions were found in the nigral neurons of chronic METH abusers, which supports the notion that UPS dysfunction may be functionally linked to METH-induced neurotoxicity [29].

Protein kinase C delta (PKC*δ*) belongs to a family of 11 structurally related serine/threonine protein kinases that play a critical role in cell proliferation, survival, and death [86]. According to recent reports, PKC*δ* is highly expressed in nigrostriatal dopaminergic neurons and the kinase is activated by proteolytic cleavage in response to oxidative stress [87]. In mesencephalic dopaminergic cell culture model, METH-induced early induction of autophagy was associated with reduction in proteasomal function and concomitant dissipation of mitochondrial membrane potential (MMP), followed by significantly increased PKC*δ* activation. Interestingly, siRNA-mediated knockdown of PKC*δ* or overexpression of cleavage-resistant mutant of PKC*δ* dramatically reduced METH-induced autophagy, proteasomal function, and associated accumulation of ubiquitinated protein aggregates, which closely paralleled cell survival [88].

Taken together, these data demonstrated that METH-induced autophagy serves as an adaptive strategy for inhibiting mitochondria-mediated apoptotic cell death and degradation of aggregated proteins. The results also suggested that the sustained activation of PKC*δ* leads to UPS dysfunction, resulting in the activation of caspase-3-mediated apoptotic cell death in the nigrostriatal dopaminergic system [88].

### 3.3. Protein Nitration

Among the most popular explanations for METH-induced neurotoxicity, it has been postulated that oxidative stress plays an essential role in the pathogenesis [89]. It is now well established that in CNS radical nitric oxide (•NO) participates as a cytotoxic effect when produced at high rates due to activation of DDAH/ADMA/NOS pathway [90, 91]. Several studies have indicated that reactive nitrogen species (RNS), the secondary intermediates of •NO, such as peroxynitrite anion (ONOO^−^) and nitrogen dioxide (•NO_2_), dramatically increase in different brain regions in human and rodents exposed to METH [30, 31].

Protein tyrosine nitration is an important posttranslational modification mediated by nitric oxide (NO) associated oxidative stress, occurring in a variety of neurodegenerative diseases [92]. In a variety of neurodegenerative diseases, such as PD and Alzheimer's (AD), protein tyrosine nitration has been indicated as a salient feature of the pathological mechanism [93, 94]. In a previous study, an elevated level of dimethylarginine dimethylaminohydrolase 1 (DDAH1) protein was observed in different brain regions of acute METH treated rats, indicating the possibility of an enhanced expression of protein nitration that was mediated by excessive NO through the DDAH1/ADMA (asymmetric dimethylated L-arginine)/NOS (nitric oxide synthase) pathway [23]. DDAH1, a major isoform of DDAH, is chiefly expressed in the nervous system [95]. Studies showed that an increased level of DDAH1 leads to excessive production of NO through DDAH1/ADMA/NOS pathway [96]. In the present study, acute METH administration evokes a positive activation of DDAH1/ADMA/NOS pathway and results in an overproduction of NO in different brain regions of rat and PC12 cells, whereas the whole signaling could be repressed by DDAH1 inhibitor N^*ω*^-(2-methoxyethyl)-arginine (L-257). In addition, enhanced expressions of 3 nitroproteins were identified in rat striatum and increased levels of 27 nitroproteins were observed in PC12 cells. These nitrated proteins are key factors for Cdk5 activation, cytoskeletal structure, ribosomes function, and so forth. L-257 also displayed significant protective effects against METH-induced protein nitration, apoptosis, and cell death [24]. The overall results illustrate that protein nitration plays a significant role in the acute METH-induced neurotoxicity via the activation of DDAH1/ADMA/NOS pathway.

### 3.4. Endoplasmic Reticulum Stress (ERS)

METH neurotoxicity is involved in METH-related deaths. It has been suggested that the midbrain, together with the striatum, is affected by METH neurotoxicity [97, 98]. The endoplasmic reticulum stress (ERS) is involved in the processes of neuronal apoptosis in the striatum of animals treated with neurotoxic METH [32, 33]. During ERS, glucose-regulated protein 78 (GRP78), one of the endoplasmic reticulum chaperone molecules, can bind unfolded proteins, degrade misfolded proteins [99], and may aid in neuroprotection [50]. C/EBP homologous protein (CHOP), a transcription factor induced under ERS, has been suggested to be involved in ERS-induced apoptosis by reducing the expression of anti-B-cell lymphoma protein 2 (Bcl-2) [100]. Increases in both GRP78 and CHOP expression were found in the striatum of mice that had received neurotoxic doses of METH [32, 33].

On the other hand, in contrast to large-dose METH, chronic human METH abusers administrate low-dose METH repeatedly over an extended period before lethal injection, investigation of the pathophysiology of METH neurotoxicity in animals pretreated with low-dose METH might provide useful information on the pathophysiology of chronic and/or lethal METH use in cases of METH-related deaths [15]. According to recent study, low-dose METH (1.0 mg/kg) pretreatment increased GRP78 levels and inhibited the induction of CHOP in the midbrain without METH neurotoxicity, compared to high-dose METH. These findings of ERS in animals pretreated with METH were associated with an early increase in SOD1 levels and upregulation of Bcl-2 [15]. Therefore, pretreatment with low-dose METH may be protective against METH neurotoxicity in the midbrain, leading to the suppression of oxidative stress and apoptotic mechanisms, in part via ERS-related pathways.

### 3.5. Expression of p53

Apoptosis-inducing transcription factor p53 is implicated in METH neurotoxicity based on the finding of attenuated METH-induced dopaminergic cell damage, especially dopaminergic terminals, in p53-knockout mice [101]. In previous report, repeated METH injections increased p53-DNA binding activity in the striatum, which was markedly attenuated in Cu, Zn-superoxide dismutase transgenic mice, but not affected by treatment with N-methyl-D-aspartate or D1 receptor antagonists [102]. These results suggested that oxidative stress-induced atrial p53-DNA binding activates downstream apoptotic pathways involved in METH neurotoxicity [103].

The p53-activated gene 608 (PAG608) was a proapoptotic gene activated and regulated by p53 expression in oxidative stress-induced apoptosis of neuronal cells [34]. PAG608 could also mediate molecular and morphological apoptotic changes produced by dopaminergic neurotoxin 6-hydroxydopamine (6-OHDA) in catecholaminergic PC12 cells by upregulation of p53 and Bax expression through its unclear and nucleolar localization [35]. Recent results showed that suppression of PAG608 using transient and stable transfection with PAG608 antisense cDNA or small interfering RNA attenuates METH-induced death of various monoaminergic neuronal cells, suggesting that METH neurotoxicity in monoaminergic cells was related, at least in part, to induction of PAG608 expression [36].

### 3.6. Inflammatory Cytokine

Cadet and colleagues reported that METH-induced neurotoxicity and gliosis were attenuated in IL-6 knockout mice [37] and that METH injection increased the expression of transcription factor NF-AT, which promotes IL-4 expression [38]. Moreover, chronic METH exposure alters immune function [39]. The latter reports imply the involvement of neuroinflammatory processes in METH-induced neurotoxicity. In conclusion, current reports showed involvement of some inflammatory molecular events in METH-induced dopaminergic neurotoxicity, such as overexpression of cyclooxygenase-2 in the striatum [40], activation of microglia [66, 104, 105], minocycline [106], tumor necrosis factor-*α* (TNF-*α*) [107], and attenuation in lack of interleukin-6 (IL-6) [37].

### 3.7. D_3_ Receptor

Recent studies have demonstrated that D_3_ receptors (D3Rs) are associated with METH addiction [41, 108, 109]. For example, a recent study showed that inhibition of the dopamine D3R attenuated the rewarding and incentive motivational effects of METH in rats [41]. A previous study showed that the D3R plays distinct roles in modulating METH-induced behavioural sensitization [110]. Therefore, D3Rs may be potential targets for the treatment of METH dependence [111, 112].

Interestingly, a recent study indicated that the D3R was involved in METH-induced hyperthermia [113]. Previous studies showed that D3R altered the immune response of activated T cells and the migration and homing of naïve CD8^+^ T cells [114]. Additionally, the thymus expresses dopamine receptors, and METH treatment altered immunocompetent cell populations in the thymus of mice [115]. Moreover, the selective modulation of cytokines by dopamine is mediated by dopamine receptors expressed on immune cells lodged in the lungs [116]. Together, these studies indicate that METH may induce alterations in the thymic and lung immune response and that dopamine receptors may be involved in this immune response.

### 3.8. Microtubule Deacetylation

METH has been increasingly recognized to impact also the blood-brain barrier (BBB), causing the release of inflammatory mediators and astrogliosis [117]. METH-induced permeability at the BBB level has been consistently reported both* in vivo *and* in vitro* [118], as a result of tight junction and cytoskeleton disarrangement [119, 120]. Recently, Fernandes et al. showed, also in endothelial cells, that exposure to METH leads to disruption of actin filaments concomitant with claudin-5 translocation to the cytoplasm, promoted by MMP-9 activation in association with ILK overexpression [42].

Similar to the actin filaments, microtubules play a critical role in cell stability and dynamics. Microtubule deacetylation is carried out by histone deacetylase (HDAC) 6, a class II HDAC, and the class III HDAC sirtuin 2 (SIRT2), which form a complex that allows them to bind to tubulin [121]. Although there are several studies showing that METH and other psychostimulants affect the expression of HDACs [122, 123], the effect of METH in microtubules acetylation was not yet explored.

## 4. Treatment of Neurotoxicity

### 4.1. Minocycline

Minocycline is a second-generation tetracycline that easily crosses the blood-brain barrier [43, 44] and it has powerful anti-inflammatory and neuroprotective properties [124–126]. Minocycline produces neuroprotective effects in several animal models of neurological diseases, including amyotrophic lateral sclerosis [45], Huntington's disease [46, 47], and Parkinson's disease [127].* In vivo* microdialysis study demonstrated that pretreatment with minocycline (40 mg/kg) significantly attenuated increased extracellular DA levels in the striatum after the administration of METH (3 mg/kg). Interestingly, METH-induced neurotoxicity in the striatum was significantly attenuated by the posttreatment and subsequent administration of minocycline (40 mg/kg) [106]. The neuroprotective effects of minocycline can occur indirectly by microglial activation and proliferation [125]. On the other hand, direct neuronal protection by minocycline has been documented, and this mode of protection is likely to be associated with the preservation of mitochondrial integrity and cytochrome c, followed by the suppression of caspase-dependent as well as caspase-independent cell death [45, 47]. Therefore, minocycline could be considered as a useful drug for the treatment of several symptoms associated with METH abuse in humans.

### 4.2. Parkin

Parkin is a ubiquitin-protein E3 ligase; its primary function is to add polyubiquitin chains to proteins destined for degradation by the 26S proteasome [128–130]. A deficit in parkin function in DA neurons leads to their neurodegeneration [131, 132]. Conversely, overexpression of parkin protects DA neurons against a variety of cellular insults* in vitro* and* in vivo*, most importantly against those involved in mediating METH neurotoxicity, such as DA-induced oxidative stress, inhibition of mitochondrial function, and impairment of the proteasome [133–135]. Parkin protects DA neuronal cell bodies in the SNc of rodents from a variety of insults including 6-hydroxydopamine [136], MPTP [137], and overexpression of proteins that self-aggregate in specific neurodegenerative disease, such as alpha-synuclein [138] and tau [139]. The protection of DAergic terminals by parkin overexpression in the SNc was demonstrated after administration of 6-hydroxydopamine [140] and in *α*-synuclein-induced neuropathology [141]. In METH-exposed rats, the increase in parkin levels attenuated METH-induced decreases in striatal tyrosine hydroxylase immunoreactivity in a dose-dependent manner, indicating that parkin can protect striatal dopaminergic terminals against METH neurotoxicity [14]. These findings suggested the importance of parkin in the functioning and maintenance of DA neurons.

### 4.3. Endocannabinoid System (ECS)

The endocannabinoid system (ECS) is an endogenous neuromodulatory system, which has been shown to participate in a broad range of functions including anxiety, depression, neurogenesis, reward, cognition, learning, and memory [142]. Converging evidence suggests that endogenous cannabinoids also play a neuroprotective role in pathological situations [143]. Indeed, administration of a toxic dose of methamphetamine alters striatal levels of endocannabinoids, the endogenous ligands of CB_1_ and CB_2_ receptors, and inhibition of the hydrolysis of these compounds prevents METH-induced reduction of tyrosine hydroxylase levels, a hallmark of dopamine terminal loss in the striatum [144]. Recent research found that METH altered the levels of the major endocannabinoids, anandamide (AEA), and 2-arachidonoyl glycerol (2-AG) in the striatum, suggesting that the ECS participated in the brain responses to METH [7]. Altogether, stimulation of ECS prior to the administration of an overdose of METH considerably reduced the neurotoxicity of the drug and highlights a protective function for the ECS against the toxicity induced by drugs and other external insults to the brain.

The prominent role of CB_2_ receptors in the neuroprotective effects of endocannabinoids against METH toxicity suggests that the effects of endocannabinoids may depend on the reduction of METH-induced neuroinflammation. Indeed, endocannabinoids modulate inflammatory responses by regulating microglia function via receptor dependent and independent mechanisms, and consequently they control the generation of cytotoxic factors as TNF-*α* [145]. Several studies have demonstrated the involvement of CB_2_ receptors in the progression/arrest of brain damage, by influencing events such as microglial cell proliferation, differentiation, and migration at neuroinflammatory lesion sites [146, 147], for example, in models of neurodegenerative diseases like Parkinson's and Huntington's diseases [148]. It is also noteworthy that CB_2_ receptor expression increases in microglial cells in case of noxious conditions associated with inflammatory events [147]. Interestingly, such inflammatory processes appear to be part of METH-induced toxicity, because this drug stimulates microglial activation in dopaminergic regions such as the striatum [149] and increases TNF-*α* levels [21]. Altogether, these observations suggest that activation of CB_2_ receptors may modulate neuroinflammatory responses induced by METH and may involve a reduction of TNF-*α* production triggered by METH [147], consequently, limiting METH-induced neurotoxicity.

### 4.4. Cytokine

Interferon-*γ* (IFN-*γ*) is an inflammatory cytokine critically involved in the pathogenesis of experimental autoimmune encephalomyelitis. Cytokines can exert a variety of effects on the CNS. Similar to IFN-*γ*, another proinflammatory reaction exhibits neuroprotective effects against METH-induced neurotoxicity [37]. Treatment of mice exposed chronically to METH resulted in a significant decrease in IFN-*γ* in splenocytes [39]. Another research result suggested that IFN-*γ* injected systemically or its related molecule protects against METH-induced neurotoxicity through intracerebral molecular pathways, while it can prevent METH-induced hyperthermia through different molecular events [48].

### 4.5. Cholecystokinin-8

Cholecystokinin (CCK), a gut-brain peptide, exerts a wide range of biological activities in the gastrointestinal tract and central nervous system (CNS). CCK-8 is involved in the regulation of feeding, pain perception, and learning and memory and possibly in the pathogenesis of anxiety and psychosis [150, 151]. It modulates the release of several neurotransmitters, such as DA and gamma-aminobutyric acid (GABA), and possibly acts as a neurotransmitter/modulator [152, 153]. Previous studies have shown that CCK-8 has antioxidative stress and anti-inflammatory effects [154, 155]. In addition, it produced neuroprotective effects in neuronal injury models. Gou et al. demonstrated that pretreatment with CCK-8 inhibited changes typically induced by repeated exposure to METH, such as hyperlocomotion, behavioral sensitization, stereotypic behavior, and dopaminergic neurotoxicity. These findings make CCK-8 a potential therapeutic agent for the treatment of multiple symptoms associated with METH abuse [49].

### 4.6. Gene Therapy

ROCK2 is a prominent target for gene therapy because its inhibition has proved to have a protective effect in various cell lines and pathophysiological conditions. ROCKs are believed to take part in PD and silencing of ROCK2 contributes to neuron protection and METH abusers were reported to relate to PD. Several molecular mechanisms have been identified that account for the role of ROCKs in apoptosis. The results show that ROCK2 is a possible gene target for therapeutics in METH-induced neurotoxicity* in vitro*, providing a foundation for further* in vivo* research [51]. The PAG608 is proapoptotic gene activated and regulated by p53 expression in oxidative stress-induced apoptosis of neuronal cells. Recent results showed that suppression of PAG608 using transient and stable transfection with PAG608 antisense cDNA or small interfering RNA attenuates methamphetamine-induced death of various monoaminergic neuronal cells, suggesting that METH neurotoxicity in monoaminergic cells is related, at least in part, to induction of PAG608 expression [50].

## 5. Discussion and Conclusion

METH is a highly addictive psychostimulant drug that acts on the central nervous system through multiple physiological pathways to cause the release of central and peripheral monoamine neurotransmitters. The highly addictive properties of this drug result in widespread psychosocial issues that include medical and legal problems, at-risk behaviors, and substantial societal costs. The acute and chronic use of METH may result in DA and 5HT release, cognitive deficits, agitation, violent behavior, anxiety, confusion, and paranoia likely resulting in part from the direct neurotoxic effects of the drug. Numerous interacting mechanisms have been established to contribute to those damages produced by METH (Table 1). These mechanisms include excitotoxicity, oxidative stress, and metabolic compromise. More recently, novel contributors to METH neurotoxicity have been identified and include UPS dysfunction, protein nitration, ERS, p53 expression, D_3_ receptor, microtubule deacetylation, the endocannabinoid system, and HIV-1 Tat protein cross amplification effects. A number of therapeutic strategies have been tried to treat dependence including drug, protein, cytokine, and gene.

## Figures and Tables

**Table 1:** A brief summary of some phenomena, mechanisms, or fundamental concepts with studies from humans or from rodents.

Phenomena or mechanisms	Model	Reference
METH and HIV-1	Human astrocytes	[16–19]
Excessive dopamine	Humans, rodents	[20–24]
UPS dysfunction	Rodents	[25–29]
Protein nitration	Human, rodents	[30, 31]
ERS	Rodents	[15, 32, 33]
Expression p53	PC12 cells	[34–36]
Inflammatory molecular	Rodents	[37–40]
D3 receptor	Rodents	[41]
Microtubule deacetylation	Endothelial cells	[42]
Minocycline	Human, rodents	[43–47]
Parkin	Rodents	[14]
Endocannabinoid system	Rodents	[21]
Cytokine	Rodents	[39, 48]
Cholecystokinin-8	Rodents	[49]
ROCK2 gene therapy	PC12 cells	[50, 51]
